# Beta-blockers after percutaneous coronary intervention for acute myocardial infarction and non-reduced left ventricular ejection fraction

**DOI:** 10.3389/fcvm.2024.1447952

**Published:** 2024-11-21

**Authors:** Jun-Chang Jeong, Jong-Il Park, Byung-Jun Kim, Hong-Ju Kim, Kang-Un Choi, Jong-Ho Nam, Chan-Hee Lee, Jang-Won Son, Jong-Seon Park, Sung-Ho Her, Ki-Yuk Chang, Tae-Hoon Ahn, Myung-Ho Jeong, Seung-Woon Rha, Hyo-Soo Kim, Hyeon-Cheol Gwon, In-Whan Seong, Kyung-Kuk Hwang, Seung-Ho Hur, Kwang-Soo Cha, Seok-Kyu Oh, Jei-Keon Chae, Ung Kim

**Affiliations:** ^1^ Division of Cardiology, Yeungnam University Medical Center, Daegu, Republic of Korea; ^2^ Division of Cardiology, Hanmaeum Medical Center, Changwon-si, Gyeongsangnam-do, Republic of Korea; ^3^ Division of Cardiology, St. Vincent’s Hospital, College of Medicine, Suwon, Republic of Korea; ^4^ Division of Cardiology, Seoul St. Mary's Hospital, College of Medicine, The Catholic University of Korea, Seoul, Republic of Korea; ^5^ Korea University Anam Hospital, Seoul, Republic of Korea; ^6^ Department of Cardiovascular Medicine, Chonnam National University Hospital, Gwangju, Republic of Korea; ^7^ Guro Hospital, Korea University, Seoul, Republic of Korea; ^8^ Department of Internal Medicine and Cardiovascular Center, Seoul National University Hospital, Seoul, Republic of Korea; ^9^ Samsung Medical Center, Sungkyunkwan University, Seoul, Republic of Korea; ^10^ Department of Cardiology, Chungnam National University Hospital, Chungnam National University School of Medicine, Daejeon, Republic of Korea; ^11^ Department of Internal Medicine, Chungbuk National University Hospital, Cheongju, Republic of Korea; ^12^ Division of Cardiology, Keimyung University Dongsan Medical Center, Daegu, Republic of Korea; ^13^ Department of Cardiology and Medical Research Institute, Pusan National University Hospital, Busan, Republic of Korea; ^14^ Department of Cardiovascular Medicine, Regional Cardiocerebrovascular Center, Wonkwang University Hospital, Iksan, Republic of Korea; ^15^ Department of Cardiology, Chonbuk National University Hospital, Jeonju, Republic of Korea

**Keywords:** beta-blockers, myocardial infarction, percutaneous coronary intervention, left ventricular ejection fraction, patient-oriented composite endpoints

## Abstract

**Background:**

Data on the clinical impact of beta-blockers (BBs) in patients with myocardial infarction (MI) who had non-reduced left ventricular ejection fraction (LVEF) after percutaneous coronary intervention are limited.

**Methods:**

From 2016 to 2020, we evaluated a cohort of 12,101 myocardial infarction patients with a non-reduced LVEF (≥40%) from the Korean Acute Myocardial Infarction Registry V. Patients were divided into two groups based on their BB (carvedilol, bisoprolol, or nebivolol) treatment at discharge: with beta-blocker treatment (BB, *n* = 9,468) and without beta-blocker treatment (non-BB, *n* = 2,633). The primary endpoint after discharge was the occurrence of patient-oriented composite endpoints (POCEs), including all-cause mortality, any MI, or any revascularization at 1-year follow-up.

**Results:**

The median follow-up period was 353 days (interquartile range, 198–378 days). At 1-year follow-up, no significant differences were observed in the primary endpoint between the BB group and the non-BB group. Before propensity score (PS) matching, the POCE incidence was 3.1% in the BB group vs. 3.4% in the non-BB group [hazard ratio (HR) 0.86, 95% confidence interval (CI) 0.68–1.09, *p* = 0.225]. After PS matching, the POCE incidence remained similar between the two groups (3.7% vs. 3.4%, HR 1.01, 95% CI 0.76–1.35, *p* = 0.931). Individual outcomes, including all-cause mortality, myocardial infarction, and revascularization, also showed no significant differences between the two groups. Independent predictors of 1-year POCEs after discharge were age, chronic kidney disease, reduced LVEF, and multivessel disease.

**Conclusion:**

BB treatment in patients with acute MI and non-reduced LVEF was not associated with a significant reduction in cardiovascular outcomes at 1-year follow-up.

## Introduction

The management of acute myocardial infarction (MI) has advanced substantially over the past decades, primarily due to the integration of reperfusion strategies and pharmacological therapies aimed at improving patient outcomes (1–7). In particular, for acute MI patients and reduced left ventricular systolic function, beta-blockers (BBs) are essential to reduce cardiovascular mortality and recurrent MI (8). Consequently, recent guidelines strongly recommend the application of BBs in patients with acute myocardial infarction and reduced left ventricular function, highlighting their critical role in improving patient outcomes (3, 4). Several studies have been conducted on using BBs in patients with acute MI and non-reduced left ventricular ejection fraction (LVEF) (9–13). However, the outcomes of these investigations remain controversial, partly due to the absence of large-scale randomized controlled trials. Consequently, current guidelines do not strongly recommend the long-term use of BBs in these patients (4). Furthermore, there is a notable lack of research on how long BB therapy should be continued in acute MI patients with non-reduced LVEF, leaving a significant gap in clinical practice guidance. Therefore, our study aimed to evaluate the clinical impact and determine the optimal duration of BB therapy in patients with acute MI and non-reduced LVEF by comparing the short-term effects (within 30 days) and 1-year outcomes, addressing this important gap in current clinical practice.

## Methods

### Study population and design

**Figure 1:**
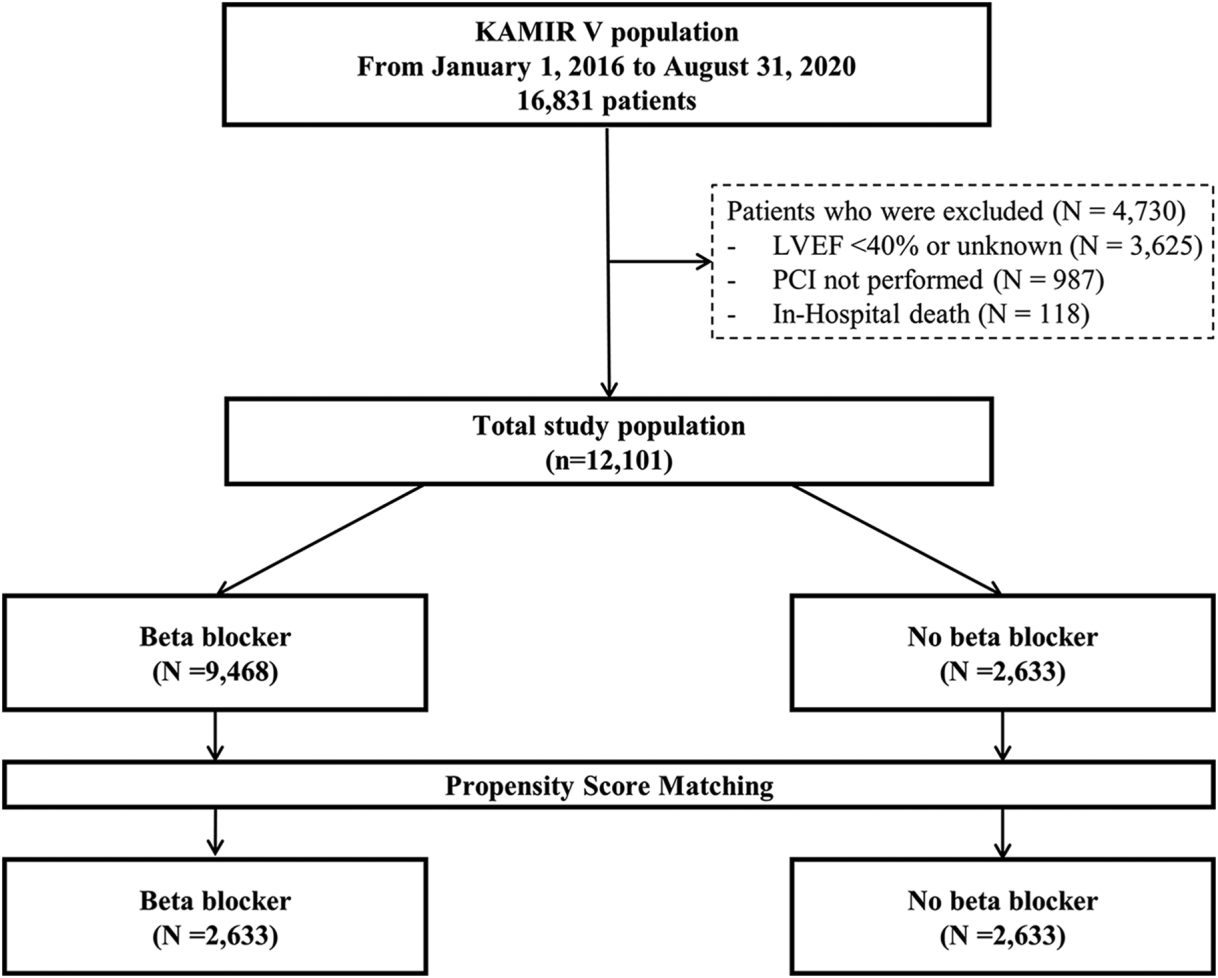
Flow diagram of the study population. KAMIR V, Korean Acute Myocardial Infarction Registry V; LVEF, left ventricular ejection fraction; PCI, percutaneous coronary intervention.

The Korean Acute Myocardial Infarction Registry V (KAMIR V) is an open, prospective, observational, web-based, nationwide, multicenter acute myocardial infarction registry from 55 primary percutaneous coronary intervention (PCI) centers started in January 2016. Trained coordinators investigated clinical, laboratory, medication, and outcome data using standardized case report forms and protocols. Angiographic data were collected and assessed by the operators. Clinical data were investigated at 1, 6, and 12 months after discharge. A total of 16,831 patients initially diagnosed with acute MI were investigated. Patients with a reduced and unknown LVEF (*n* = 3,625) and those who did not undergo PCI (*n* = 987) were excluded. In addition, 118 patients who died in the hospital were excluded, leaving a total of 12,101 patients in this study. Then, participants were categorized into two groups based on the presence or absence of BB treatment at discharge, as BB use prior to discharge was not documented: the BB group (*n* = 9,468) and the non-BB group (*n* = 2,633). The flow diagram of this study is shown in Figure 1. This study followed the guidelines of the Declaration of Helsinki and was approved by the institutional review board of Yeungnam University Medical Center (YUHIRB 2016-03-017). This study was conducted after obtaining informed consent from the patients after a thorough explanation.

### Study endpoints and definitions

We defined non-reduced LVEF as a concept in contrast with heart failure (HF) with reduced ejection fraction. The primary endpoint of this study was the occurrence of patient-oriented composite endpoints (POCEs), including all-cause mortality, any MI, and any revascularization, at 1-year follow-up after PCI. The secondary endpoints of this study consisted of each component of the POCE during 1-year follow-up. Chronic kidney disease (CKD) was defined as either a pre-existing diagnosis or a serum creatinine level >1.5 mg/dl. Complex PCI was defined as PCI involving the left main artery, an implanted stent length ≥38 mm, PCI for multivessel disease, or implantation of ≥3 stents (14).

### Statistical analysis

Categorical data were presented as absolute numbers and percentages and analyzed using the *χ*^2^ statistic or Fisher’s exact test to compare variables. Continuous data were expressed as mean ± standard deviation and compared using Student’s *t*-test. Survival curves were generated using the Kaplan–Meier method and compared with the log-rank test. A landmark analysis at 1 month was performed to compare the early and late effects of BB treatment. A propensity score (PS) for treatment with BB was calculated using multiple logistic regression analysis incorporating variables such as age, sex, hypertension, diabetes mellitus, dyslipidemia, heart failure, CKD, clinical presentation [ST-elevation myocardial infarction (STEMI) or non-ST-elevation myocardial infarction (NSTEMI)], complex PCI, and successful PCI that could affect clinical outcomes and treatment group assignment. PS matching was performed using a 1:1 case–control match on the PS with a hierarchical sequence until no more matches could be made. This was done to minimize selection bias and adjust for confounding factors resulting from significant differences in baseline and procedural characteristics between the two groups. In the univariate analysis, a Cox proportional hazards regression model was conducted for several clinical variables. Those variables achieving a *p*-value <0.1 in the univariate analysis were subsequently entered into the multivariate analysis model to determine independent predictors of POCEs. Event-free survival was analyzed using Kaplan–Meier survival curves, and differences between event-free survival curves were compared using the log-rank test. All hazard ratios (HRs) were calculated with a 95% confidence interval (CI), and *p*-values <0.05 were considered statistically significant. The proportional hazard assumption for the Cox regression model was tested using log(-log) survival plots and Schoenfeld residuals, with a *p*-value >0.05 indicating no violation of the assumption. Statistical analysis was implemented using R 4.4.1.

## Results

### Baseline and angiographic characteristics

The mean age was 63.3 ± 12.2 years, with 78.8% of the patients being men. Of the 12,101 patients with non-reduced LVEF, 9,468 (78.2%) received BBs at discharge, and 60.4% remained on BB therapy at 1-year follow-up. Table 1 presents the baseline characteristics of the BB and non-BB groups. Compared to the non-BB group, the BB group was younger and exhibited a lower LVEF, with a lower prevalence of previous angina pectoris, HF, atrioventricular block, cardiogenic shock, and NSTEMI. However, the BB group had a higher prevalence of hypertension, higher initial systolic/diastolic blood pressure, a higher rate of current smoking, and a greater incidence of STEMI. In terms of laboratory findings at admission, the BB group exhibited lower serum N-terminal pro-B-type natriuretic peptide levels and higher levels of serum total cholesterol, low-density lipoprotein cholesterol, and triglycerides compared to the non-BB group.

**Table 1:** Baseline characteristics.

Characteristics	Before propensity score matching (*N* = 12,101)			After propensity score matching (*N* = 5,266)		
	Beta-blockers (*N* = 9,468)	No beta-blockers (*N* = 2,633)	*p*-value	Beta-blockers (*N* = 2,633)	No beta-blockers (*N* = 2,633)	*p*-value
Age (years)	62.9 ± 12.2	64.7 ± 12.0	<0.001	65.1 ± 12.4	64.7 ± 12.0	0.174
Female patient, *n* (%)	1,983 (20.9)	587 (22.3)	0.141	624 (23.7)	587 (22.3)	0.238
Systolic blood pressure (mmHg)	116.9 ± 15.2	115.7 ± 15.5	<0.001	117.0 ± 15.3	115.7 ± 15.5	0.004
Diastolic blood pressure (mmHg)	70.0 ± 10.2	69.0 ± 10.4	<0.001	69.4 ± 10.1	69.0 ± 10.4	0.194
Hypertension, *n* (%)	4,668 (49.3)	1,203 (45.7)	0.001	1,220 (46.3)	1,203 (45.7)	0.658
Diabetes mellitus, *n* (%)	2,510 (26.5)	654 (24.8)	0.089	700 (26.6)	654 (24.8)	0.156
Dyslipidemia, *n* (%)	1,370 (14.5)	385 (14.6)	0.869	463 (17.6)	385 (14.6)	0.004
Previous MI, *n* (%)	528 (5.6)	168 (6.4)	0.129	173 (6.6)	168 (6.4)	0.823
History of angina pectoris, *n* (%)	620 (6.5)	232 (8.8)	<0.001	268 (10.2)	232 (8.8)	0.100
History of heart failure, *n* (%)	53 (0.6)	27 (1.0)	0.013	25 (0.9)	27 (1.0)	0.889
History of CVA, *n* (%)	573 (6.1)	159 (6.0)	1.000	162 (6.2)	159 (6.0)	0.908
Chronic kidney disease, *n* (%)	608 (6.4)	190 (7.2)	0.159	194 (7.4)	190 (7.2)	0.874
Atrial fibrillation, *n* (%)	403 (4.3)	111 (4.2)	0.970	127 (4.8)	111 (4.2)	0.320
Current smoker, *n* (%)	3,791 (40.0)	998 (37.9)	0.050	954 (36.2)	998 (37.9)	0.220
Clinical diagnosis			<0.001			0.696
STEMI, *n* (%)	4,817 (50.9)	1,122 (42.6)		1,107 (42.0)	1,122 (42.6)	
NSTEMI, *n* (%)	4,651 (49.1)	1,511 (57.4)		1,526 (58.0)	1,511 (57.4)	
LVEF (%)	54.8 ± 8.4	55.5 ± 8.7	0.001	55.5 ± 8.7	55.5 ± 8.7	0.717
Atrioventricular block, *n* (%)	57 (0.6)	44 (1.7)	<0.001	33 (1.3)	44 (1.7)	0.251
Cardiogenic shock, *n* (%)	276 (2.9)	102 (3.9)	0.015	100 (3.8)	102 (3.9)	0.943
IABP, *n* (%)	59 (0.6)	16 (0.6)	1.000	13 (0.5)	16 (0.6)	0.710
ECMO, *n* (%)	24 (0.3)	8 (0.3)	0.818	15 (0.6)	8 (0.3)	0.210
Laboratory findings						
Total cholesterol (mg/dl)	180.1 ± 54.6	174.5 ± 45.7	<0.001	179.5 ± 54.4	174.5 ± 45.7	0.001
LDL (mg/dl)	113.9 ± 40.4	109.5 ± 39.1	<0.001	114.0 ± 43.8	109.5 ± 39.1	<0.001
HDL (mg/dl)	43.7 ± 11.8	43.5 ± 12.5	0.604	44.5 ± 12.7	43.5 ± 12.5	0.013
TG (mg/dl)	152.0 ± 136.3	143.4 ± 107.4	0.002	154.2 ± 166.8	143.4 ± 107.4	0.010
Hemoglobin (g/dl)	14.1 ± 2.0	13.9 ± 1.9	<0.001	13.9 ± 2.0	13.9 ± 1.9	0.880
Creatinine (mg/dl)	1.1 ± 1.0	1.1 ± 1.3	0.039	1.1 ± 1.2	1.1 ± 1.3	0.632
hsCRP (mg/dl)	1.8 ± 10.0	2.2 ± 10.1	0.186	2.2 ± 10.3	2.2 ± 10.1	0.994
Peak TnI (ng/ml)	49.8 ± 292.1	58.3 ± 354.2	0.354	44.6 ± 301.5	58.3 ± 354.2	0.210
Peak CK-MB (ng/ml)	109.2 ± 140.8	107.2 ± 144.0	0.539	116.4 ± 126.2	107.2 ± 144.0	0.015
NT-proBNP (pg/ml)	1,411.1 ± 4,300.6	1,780.6 ± 5,129.3	0.015	1,636.5 ± 4,838.0	1,780.6 ± 5,129.3	0.422
Medications						
Aspirin, *n* (%)	9,449 (99.8)	2,605 (98.9)	<0.001	2,628 (99.8)	2,605 (98.9)	<0.001
P2Y12 inhibitors, *n* (%)	9,443 (99.7)	2,597 (98.6)	<0.001	2,626 (99.7)	2,597 (98.6)	<0.001
RAS blockers, *n* (%)	7,599 (80.3)	1,584 (60.2)	<0.001	2,148 (81.6)	1,584 (60.2)	<0.001
CCB, *n* (%)	577 (6.1)	470 (17.9)	<0.001	171 (6.5)	470 (17.9)	<0.001
Statin, *n* (%)	9,148 (96.6)	2,472 (93.9)	<0.001	2,559 (97.2)	2,472 (93.9)	<0.001
Ezetimibe, *n* (%)	960 (10.1)	204 (7.7)	<0.001	452 (17.2)	204 (7.7)	<0.001
GP IIb/IIIa inhibitors, *n* (%)	1,085 (11.5)	244 (9.3)	0.002	245 (9.3)	244 (9.3)	1.000
Anticoagulants, *n* (%)	379 (4.0)	88 (3.3)	0.134	99 (3.8)	88 (3.3)	0.457

MI, myocardial infarction; CVA, cerebrovascular accident; NSTEMI, non-ST-elevation myocardial infarction; STEMI, ST-elevation myocardial infarction; LVEF, left ventricular ejection fraction; AVB, atrioventricular block; IABP, intra-aortic balloon pump; ECMO, extracorporeal membrane oxygenation; LDL, low-density lipoprotein; HDL, high-density lipoprotein; TG, triglyceride; hsCRP, high-sensitivity C-reactive protein; TnI, troponin I; CK-MB, creatine kinase muscle brain; NT-proBNP, N-terminal pro-B-type natriuretic peptide; RAS, renin–angiotensin system; CCB, calcium channel blocker; GP, glycoprotein.

Table 2 presents the angiographic characteristics of the two groups. Compared to the non-BB group, the BB group had higher rates of involvement in the left anterior descending artery, multivessel disease, lesion type C as per the American College of Cardiology/American Heart Association classification, complex PCI, and successful PCI.

**Table 2:** Angiographic characteristics.

Characteristics	Before propensity score matching (*N* = 12,101)			After propensity score matching (*N* = 5,266)		
	Beta-blockers (*N* = 9,468)	No beta-blockers (*N* = 2,633)	*p*-value	Beta-blockers (*N* = 2,633)	No beta-blockers (*N* = 2,633)	*p*-value
Target vessel			<0.001			<0.001
LM, *n* (%)	219 (2.3)	57 (2.2)		68 (2.6)	57 (2.2)	
LAD, *n* (%)	4,433 (46.8)	981 (37.3)		1,197 (45.5)	981 (37.3)	
LCx, *n* (%)	1,710 (18.1)	486 (18.5)		502 (19.1)	486 (18.5)	
RCA, *n* (%)	3,061 (32.3)	1,080 (41.0)		851 (32.3)	1,080 (41.0)	
Multivessel disease, *n* (%)	3,858 (40.7)	987 (37.5)	0.003	1,097 (41.7)	987 (37.5)	0.002
Lesion type C according to the ACC/AHA classification, *n* (%)	4,413 (46.6)	1,095 (41.6)	<0.001	1,353 (51.4)	1,095 (41.6)	<0.001
Unprotected LM PCI, *n* (%)	327 (3.5)	95 (3.6)	0.748	92 (3.5)	95 (3.6)	0.882
Complex PCI^a^, *n* (%)	3,980 (42.0)	1,027 (39.0)	0.006	1,121 (42.6)	1,027 (39.0)	0.009
Total stent number, *n*	1.4 ± 0.9	1.4 ± 0.9	0.081	1.3 ± 0.8	1.4 ± 0.9	0.034
Total stent length (mm)	30.9 ± 15.2	30.1 ± 14.4	0.015	31.3 ± 14.9	30.1 ± 14.4	0.004
Stent diameter of the target lesion (mm)	3.1 ± 0.5	3.1 ± 0.5	0.400	3.2 ± 0.5	3.1 ± 0.5	<0.001
Stent length of the target lesion (mm)	25.6 ± 7.7	25.3 ± 7.7	0.139	26.1 ± 7.6	25.3 ± 7.7	<0.001
IVUS, *n* (%)	2,269 (24.0)	588 (22.3)	0.086	833 (31.6)	588 (22.3)	<0.001
Tx strategy of STEMI			0.401			0.234
Primary PCI, *n* (%)	4,739 (98.4)	1,111 (99.0)		1,102 (99.5)	1,111 (99.0)	
Elective PCI, *n* (%)	59 (1.2)	9 (0.8)		3 (0.3)	9 (0.8)	
Thrombolysis, *n* (%)	14 (0.3)	1 (0.1)		0 (0)	1 (0.1)	
Conservative Tx, *n* (%)	5 (0.1)	1 (0.1)		2 (0.2)	1 (0.1)	
Tx strategy of NSTEMI			0.038			0.043
Early invasive PCI, *n* (%)	3,666 (78.8)	1,152 (76.2)		1,211 (79.4)	1,152 (76.2)	
No early invasive PCI, *n* (%)	985 (21.2)	359 (23.8)		315 (20.6)	359 (23.8)	
Successful PCI, *n* (%)	9,317 (98.4)	2,572 (97.7)	0.016	2,572 (97.7)	2,572 (97.7)	1.000

LAD, left anterior descending artery; LCx, left circumflex artery; RCA, right coronary artery; LM, left main; ACC/AHA, American College of Cardiology/American Heart Association; PCI, percutaneous coronary intervention; IVUS, intravascular ultrasound; Tx, treatment; STEMI, ST-elevation myocardial infarction; NSTEMI, non-ST-elevation myocardial infarction.

^a^ Complex PCI was defined as PCI for LM disease, multivessel disease, ≥2 vessels treated during one PCI session, and ≥3 stents implanted.

In summary, the BB group exhibited an unfavorable clinical profile and more complex lesions in the coronary artery compared to the non-BB group. To address significant differences in baseline and angiographic characteristics, PS matching was performed. After PS matching, baseline, and angiographic characteristics were well balanced.

### Prescription pattern of beta-blockers

In the total population, carvedilol (45.1%) was the most commonly prescribed BB at discharge, followed by bisoprolol (29.6%), nebivolol (24.5%), and other BBs (0.8%) (Supplementary Figure S1).

### Clinical endpoints according to the use of beta-blockers

The median follow-up period was 353 days (interquartile range: 194–378 days).

**Figure 2:**
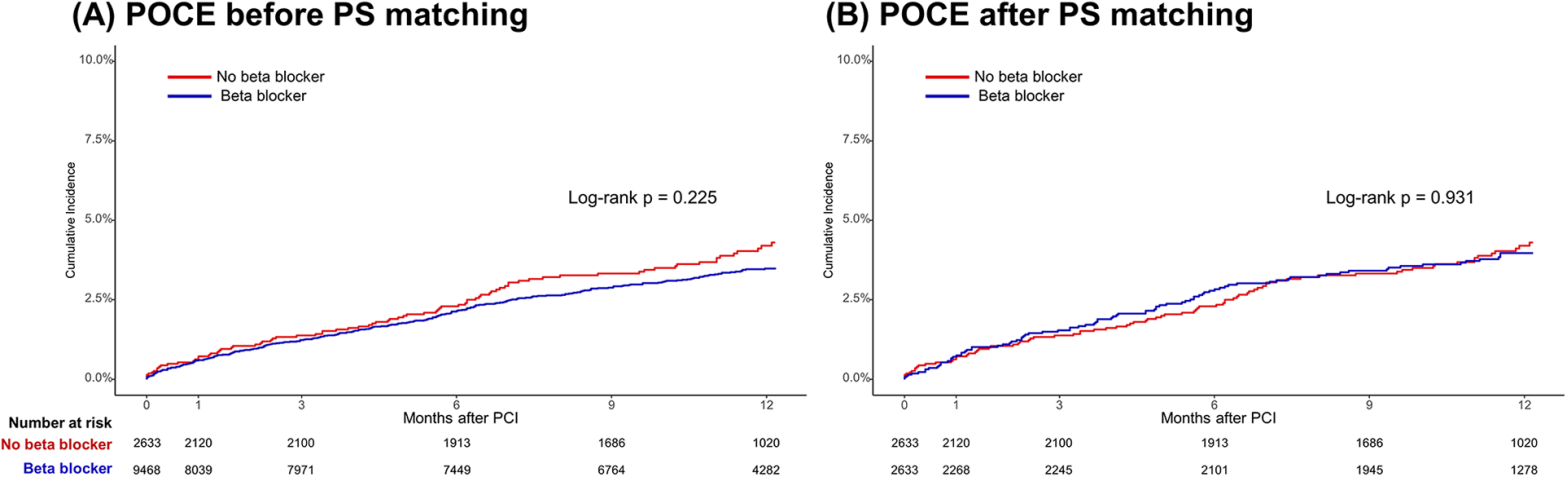
Time-to-event curves for the primary endpoint according to beta-blocker treatment. Kaplan–Meier survival curves for POCEs at the 1-year follow-up **(A)** before propensity score matching and **(B)** after propensity score matching. POCEs, patient-oriented composite endpoints.

**Figure 3:**
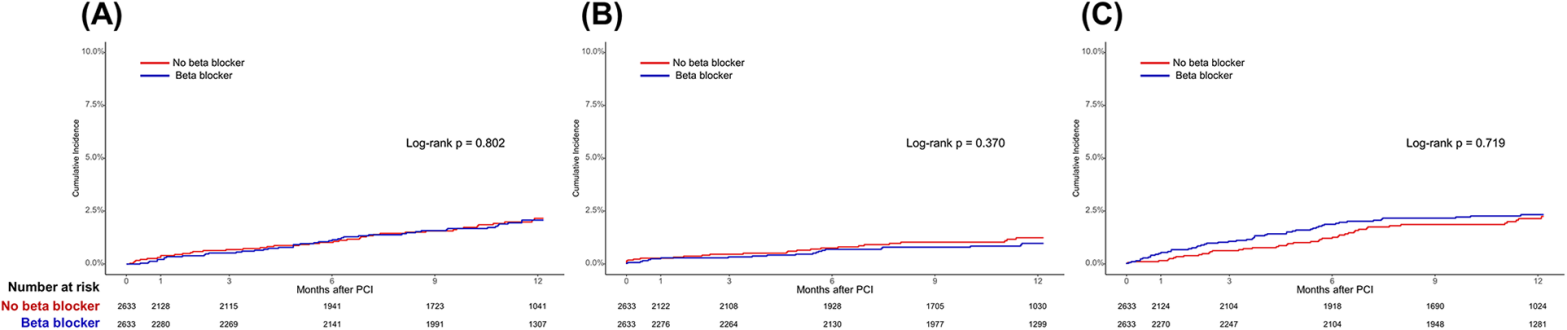
Time-to-event curves for the secondary endpoints according to beta-blocker use in the propensity score-matched population. Kaplan–Meier survival curves for **(A)** all-cause mortality, **(B)** any myocardial infarction, and **(C)** any revascularization. PCI, percutaneous coronary intervention.

In the total population, at 1-year follow-up, there were no significant differences in POCEs between the BB and non-BB groups (3.1% vs. 3.4%, HR 0.86, 95% CI 0.68–1.09, *p* = 0.225). After PS matching, the occurrence of POCEs remained similar between the BB and non-BB groups (3.7% vs. 3.4%, HR 1.01, 95% CI 0.76–1.35, *p* = 0.931). No significant differences were observed between the BB and non-BB groups for individual endpoints, including all-cause mortality, any MI, and any revascularization, both before and after PS matching (Table 3 and Figures 2, 3). In the short-term outcomes within 1 month, no notable differences were found between the BB and non-BB groups regarding the primary endpoint, and the proportional hazard assumption was confirmed as not violated (*p* = 0.25), allowing for analysis without separating the first month from subsequent months (Supplementary Figure S2). However, there was a slight difference in the incidence of any revascularization, with the BB group showing a higher incidence (HR 3.11, 95% CI 1.01–9.54, *p* = 0.047). Importantly, in-hospital events, such as aggravated HF, ventricular tachycardia, and ventricular fibrillation, also showed no significant differences between the two groups.

**Table 3:** Clinical outcomes at 1-year follow-up and before 1-month follow-up based on beta-blocker use.

	Before PSM (*N* = 12,101)				After PSM (*N* = 5,266)			
	BB (*n* = 9,468)	Non-BB (*n* = 2,633)	HR (95% CI)	*p-*value	BB (*n* = 2,633)	Non-BB (*n* = 2,633)	HR (95% CI)	*p*-value
1-year follow-up								
Primary endpoint (POCE), *n* (%)	295 (3.1)	89 (3.4)	0.86 (0.68–1.09)	0.225	97 (3.7)	89 (3.4)	1.01 (0.76–1.35)	0.931
Composite of all-cause mortality, any myocardial infarction, or any revascularization								
Individual endpoints								
All-cause mortality, *n* (%)	143 (1.5)	43 (1.6)	0.86 (0.61–1.22)	0.402	50 (1.9)	43 (1.6)	1.05 (0.70–1.58)	0.802
Cardiac death, *n* (%)	71 (0.7)	23 (0.9)	0.80 (0.50–1.28)	0.352	26 (1.0)	23 (0.9)	1.03 (0.59–1.81)	0.916
Non-cardiac death, *n* (%)	72 (0.8)	20 (0.8)	0.94 (0.57–1.54)	0.800	24 (0.9)	20 (0.8)	1.08 (0.60–1.95)	0.803
Any MI, *n* (%)	105 (1.1)	28 (1.1)	0.98 (0.65–1.49)	0.922	23 (0.9)	28 (1.1)	0.78 (0.45–1.35)	0.372
Any revascularization, *n* (%)	178 (1.9)	50 (1.9)	0.93 (0.68–1.27)	0.631	57 (2.2)	50 (1.9)	1.07 (0.73–1.57)	0.719
Follow-up before 1 month								
Primary endpoint (POCE), *n* (%)	52 (0.5)	17 (0.6)	0.82 (0.48–1.43)	0.490	18 (0.7)	17 (0.6)	1.02 (0.52–1.97)	0.965
Composite of all-cause mortality, any myocardial infarction, or any revascularization								
Individual endpoints								
All-cause mortality, *n* (%)	19 (0.2)	9 (0.3)	0.57 (0.26–1.25)	0.159	5 (0.2)	9 (0.3)	0.52 (0.18–1.56)	0.247
Cardiac death, *n* (%)	12 (0.1)	6 (0.2)	0.53 (0.20–1.42)	0.210	4 (0.2)	6 (0.2)	0.62 (0.18–2.21)	0.466
Non-cardiac death, *n* (%)	7 (0.1)	3 (0.1)	0.63 (0.16–2.43)	0.501	1 (0.0)	3 (0.1)	0.32 (0.03–3.08)	0.324
Any MI, *n* (%)	26 (0.3)	7 (0.3)	1.01 (0.44–2.32)	0.988	7 (0.3)	7 (0.3)	0.97 (0.34–2.76)	0.952
Any revascularization, *n* (%)	23 (0.2)	4 (0.2)	1.54 (0.53–4.45)	0.425	13 (0.5)	4 (0.2)	3.11 (1.01–9.54)	0.047
In-hospital aggravated HF, *n* (%)	122 (1.3)	36 (1.4)	0.94 (0.65–1.36)	0.737	51 (1.9)	36 (1.4)	1.40 (0.91–2.14)	0.126
In-hospital VT needing anti-arrhythmic drug, *n* (%)	133 (1.4)	37 (1.4)	1.23 (0.64–2.39)	0.531	42 (1.6)	37 (1.4)	1.26 (0.58–2.72)	0.561
In-hospital sustained VT needing D/C shock, *n* (%)	103 (1.1)	28 (1.1)	1.03 (0.68–1.56)	0.903	35 (1.3)	28 (1.1)	1.26 (0.77–2.07)	0.365
In-hospital ventricular fibrillation, *n* (%)	155 (1.6)	31 (1.2)	1.40 (0.95–2.05)	0.090	32 (1.2)	31 (1.2)	1.04 (0.63–1.70)	0.893

PSM, propensity score matching; BB, beta-blocker; HR, hazard ratio; CI, confidence interval; POCE, patient-oriented composite endpoint; MI, myocardial infarction; HF, heart failure; VT, ventricular tachycardia; D/C, direct current.

### Clinical outcomes of different beta-blockers

In the total population, there were no statistically significant differences in the 1-year primary endpoint based on the type of BB used (Supplementary Table S1).

### Subgroup analysis for the primary endpoint in the PS-matched cohort

**Figure 4:**
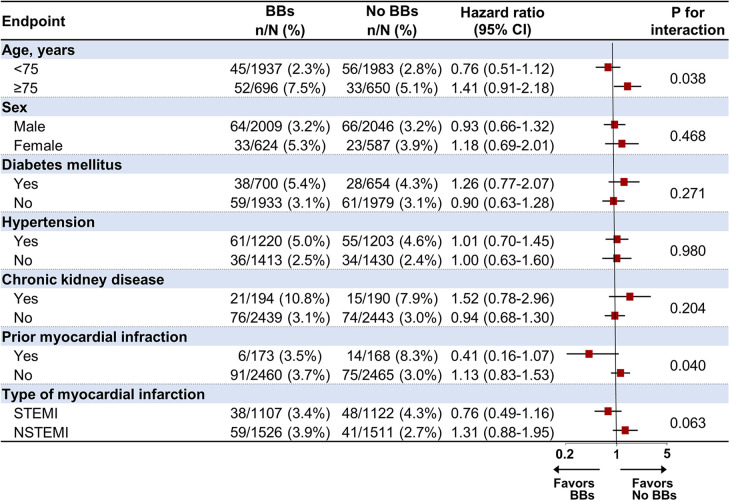
Subgroup analysis for the primary endpoint at 1-year follow-up in the propensity score-matched population. BB, beta-blocker; CI, confidence interval; STEMI, ST-elevation myocardial infarction; NSTEMI, non-ST-elevation myocardial infarction.

The association between the use of BBs and POCEs at 1-year follow-up was analyzed in prespecified subgroups, including age (<75 and ≥75), sex, hypertension, diabetes mellitus, CKD, prior MI, and type of MI (Figure 4). In the subgroup analysis, BB treatment was significantly unfavorable in patients aged 75 and older than in those under 75 (*p* for interaction = 0.038). In addition, BB treatment appeared more beneficial in patients with a history of MI, significantly lowering the risk of POCEs (HR 0.41, 95% CI 0.16–1.07) compared to those without prior MI (HR 1.13, 95% CI 0.83–1.53, *p* for interaction = 0.040). On the other hand, no significant interactions were found based on sex, diabetes mellitus, hypertension, CKD, or type of MI (STEMI/NSTEMI), suggesting that the effects of BB treatment were relatively consistent across these subgroups.

### Independent predictors for POCEs at 1-year follow-up

Table 4 presents the independent predictors for POCEs at 1-year clinical follow-up. After adjusting for confounding factors, the independent predictors for POCEs were age (HR 1.02, 95% CI 1.01–1.03, *p* < 0.001), CKD (HR 2.47, 95% CI 1.89–3.25, *p* < 0.001), LVEF (HR 0.98, 95% CI 0.97–0.99, *p* = 0.002), and multivessel disease (HR 1.57, 95% CI 1.27–1.93, *p* < 0.001). The prescription of BB at discharge (HR 0.86, 95% CI 0.68–1.09, *p* = 0.225) was not associated with a statistically significant reduction in POCEs.

**Table 4:** Independent predictors of POCEs at 1-year follow-up.

	Univariate HR (95% CI)	*p*-value	Multivariate HR (95% CI)	*p*-value
Age	1.04 (1.03–1.05)	<0.001	1.02 (1.01–1.03)	<0.001
Female patient	1.52 (1.22–1.90)	<0.001	1.08 (0.85–1.37)	0.549
Hypertension	1.73 (1.41–2.13)	<0.001	1.18 (0.94–1.47)	0.150
Diabetes mellitus	1.55 (1.26–1.91)	<0.001	1.06 (0.85–1.33)	0.617
Dyslipidemia	0.87 (0.65–1.17)	0.368		
Previous MI	1.79 (1.27–2.52)	<0.001	1.32 (0.93–1.87)	0.125
Old CVA	2.07 (1.51–2.82)	<0.001	1.34 (0.98–1.85)	0.069
Chronic kidney disease	3.67 (2.86–4.72)	<0.001	2.47 (1.89–3.25)	<0.001
Atrial fibrillation	1.98 (1.37–2.86)	<0.001	1.45 (1.00–2.11)	0.051
Current smoker	0.55 (0.44–0.69)	<0.001	0.82 (0.64–1.06)	0.125
STEMI	0.84 (0.69–1.03)	0.092	0.98 (0.79–1.21)	0.835
LVEF	0.97 (0.96–0.98)	<0.001	0.98 (0.97–0.99)	0.002
Multivessel disease	1.85 (1.51–2.26)	<0.001	1.57 (1.28–1.93)	<0.001
Left main PCI	1.66 (1.07–2.58)	0.023	1.18 (0.75–1.84)	0.476
Lesion type C according to the ACC/AHA classification	1.01 (0.83–1.24)	0.892		
IVUS	1.12 (0.89–1.41)	0.337		
RAS inhibitor	0.95 (0.75–1.20)	0.652		
Beta-blocker	0.86 (0.68–1.09)	0.225		

POCE, patient-oriented composite endpoint; HR, hazard ratio; CI, confidence interval; MI, myocardial infarction; CVA, cerebrovascular accident; STEMI, ST-elevation myocardial infarction; LVEF, left ventricular ejection fraction; PCI, percutaneous coronary intervention; ACC/AHA, American College of Cardiology/American Heart Association; IVUS, intravascular ultrasound; RAS, renin–angiotensin system.

## Discussion

The main findings of this study are as follows: (1) in this study, BB treatment at discharge was not associated with a significant difference in the POCE incidence in the PS-matched population at 1-year follow-up (3.7% vs. 3.4%, HR 1.01, 95% CI 0.76–1.35, *p* = 0.931); (2) in the short-term outcomes within 1 month, there were no notable differences between the BB and non-BB groups for the primary endpoint; and (3) independent predictors of POCEs at 1-year follow-up included age, CKD, multivessel disease, and reduced LVEF. BB use was not found to be a significant independent predictor in the multivariate analysis.

BBs have been foundational in managing acute MI, significantly benefiting patients with reduced LVEF by reducing mortality and preventing arrhythmias. The efficacy of BBs in patients with reduced LVEF is well-documented and largely undisputed, forming the basis for strong recommendations in clinical guidelines (4, 7). However, the benefit of BB treatment in patients with non-reduced LVEF, particularly in the era of advanced reperfusion therapies, remains less clear. Recently, the efficacy of BB therapy in acute MI patients with non-reduced LVEF has been extensively studied, yielding mixed outcomes. In several studies, BB therapy after MI has been found to be associated with reduced mortality and cardiovascular benefits in patients with acute MI without LV dysfunction (11, 15, 16). Conversely, in a recent prospective randomized study, the effect of beta-blockers in patients with preserved LVEF (≥50%) after MI was evaluated. The results showed that long-term beta-blocker treatment, with a median follow-up of 3.5 years, did not reduce the risk of all-cause mortality or new MI (17). In the previous randomized controlled trial of carvedilol usage in STEMI patients with non-reduced LVEF, this study did not demonstrate a significant benefit of long-term carvedilol use in these patients (10). Although this study conducted a randomized controlled trial design, the number of enrolled participants was relatively small, with a total of only 801 participants. In a nationwide cohort study involving over 30,000 MI patients without HF, no long-term effect of BB treatment on cardiovascular prognosis was observed when following the patients from 3 months to 3 years after MI admission (12). This finding aligned with the results of our study, which indicated no significant difference in 1-year outcomes between the BB and non-BB groups, suggesting that there may also be no differences in long-term outcomes.

In addition, the treatment of BBs and the duration of their usage have not been clearly studied. Two nationwide cohort studies have demonstrated that in patients without LV dysfunction undergoing PCI for acute MI, the use of BB from the first year onward did not improve cardiovascular outcomes (18, 19). Contrary to previous studies, research indicates that early use of BBs in acute MI patients without LV dysfunction could improve mortality rates at 1 month compared to non-BB users (9). Furthermore, in a larger cohort of patients with acute coronary syndrome without LV dysfunction, an initial reduction in mortality was observed at 1 month for those treated with BBs (20). However, this difference in mortality did not persist at 6 or 12 months. In contrast to earlier studies suggesting benefits from the early use of BBs, recent randomized trials have similarly shown no significant changes in outcomes within the first month or beyond 1 year (17). These findings are consistent with the results of our study, where no early (within 1 month) or long-term differences in cardiovascular outcomes were observed between the BB and non-BB groups. These results suggested that the routine use of BBs in patients with acute MI and non-reduced LVEF may not be essential. In both short-term (within 1 month) and long-term follow-ups, no notable advantages in cardiovascular outcomes were observed between those who received BBs and those who did not. This raised a question about the necessity of universally prescribing BBs in this patient population. However, while our findings aligned with recent randomized trials, further studies with longer follow-up periods are needed to fully assess the long-term impact of BB therapy in these patients.

## Limitations

This study has several limitations. First, its retrospective observational nature may introduce inherent biases. One key limitation is that, in the acute stage, patients who were more severely ill may have been less likely to receive beta-blockers due to their critical condition, which may have precluded the use of such medications. While we applied PS matching and multivariate analysis to adjust for these confounders, these statistical techniques may not have been sufficient to fully account for the baseline differences, potentially leading to an overestimation of the favorable association between early beta-blocker use and improved outcomes, especially in the non-beta-blocker group. Second, while we investigated BB prescription at discharge and its adherence during 1-year follow-up, the actual adherence to BB treatment and the exact duration of BB usage could not be definitively determined in this study. Third, there was no guarantee that the BB prescription at discharge aligned perfectly with the BB prescription at admission. Despite these limitations, this study offers significant advantages owing to its larger population size and baseline characteristics, which are more consistent with the current revascularization era compared to previous studies. Fourth, the relatively short follow-up duration may have limited our ability to fully assess the long-term effects of beta-blockers in this subset of patients. Further long-term clinical studies will be needed in patients with non-reduced LVEF.

## Conclusion

In conclusion, in patients with acute MI and non-reduced LVEF, beta-blocker treatment at discharge was not associated with a significant difference in clinical cardiovascular outcomes, either within the first 30 days or at 1-year follow-up. Given the need for a more comprehensive evaluation of beta-blocker therapy, longer follow-up beyond 1 year is required to fully assess its long-term effects, warranting further investigation.

## Data Availability

The raw data supporting the conclusions of this article will be made available by the authors without undue reservation.
