# Supplementary material for: Beta-blockers after percutaneous coronary intervention for acute myocardial infarction and non-reduced left ventricular ejection fraction
Source: Front Cardiovasc Med. 2024 Nov 21;11:1447952. doi: 10.3389/fcvm.2024.1447952 (PMC11617510; doi:10.3389/fcvm.2024.1447952)
Supplement: Supplementary file 1 [file Datasheet1.docx]

**Beta-Blockers after Percutaneous Coronary Intervention for Acute Myocardial Infarction and Non-reduced Left Ventricular Ejection Fraction**

**Supplementary Figure 1. Prescription rate by type of beta-blockers**

**
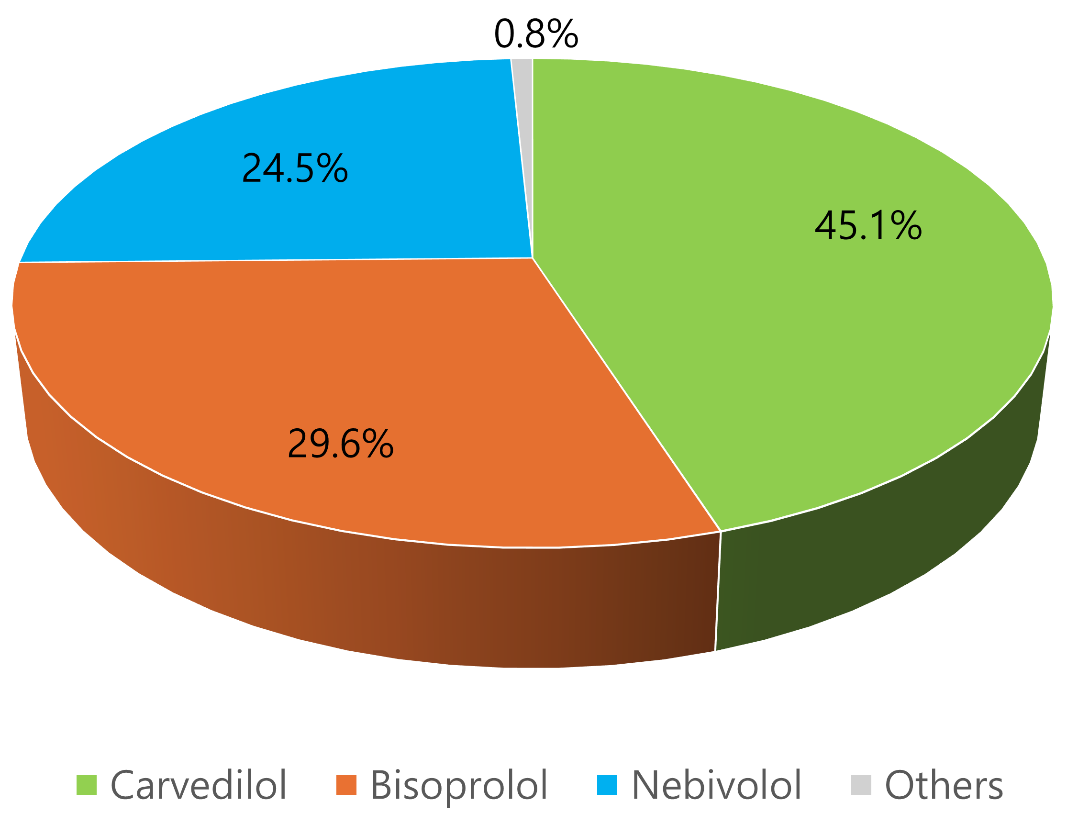
**

**Supplementary Figure 2. Log-log Kaplan-Meier curves for POCE before and after 1 month**

**
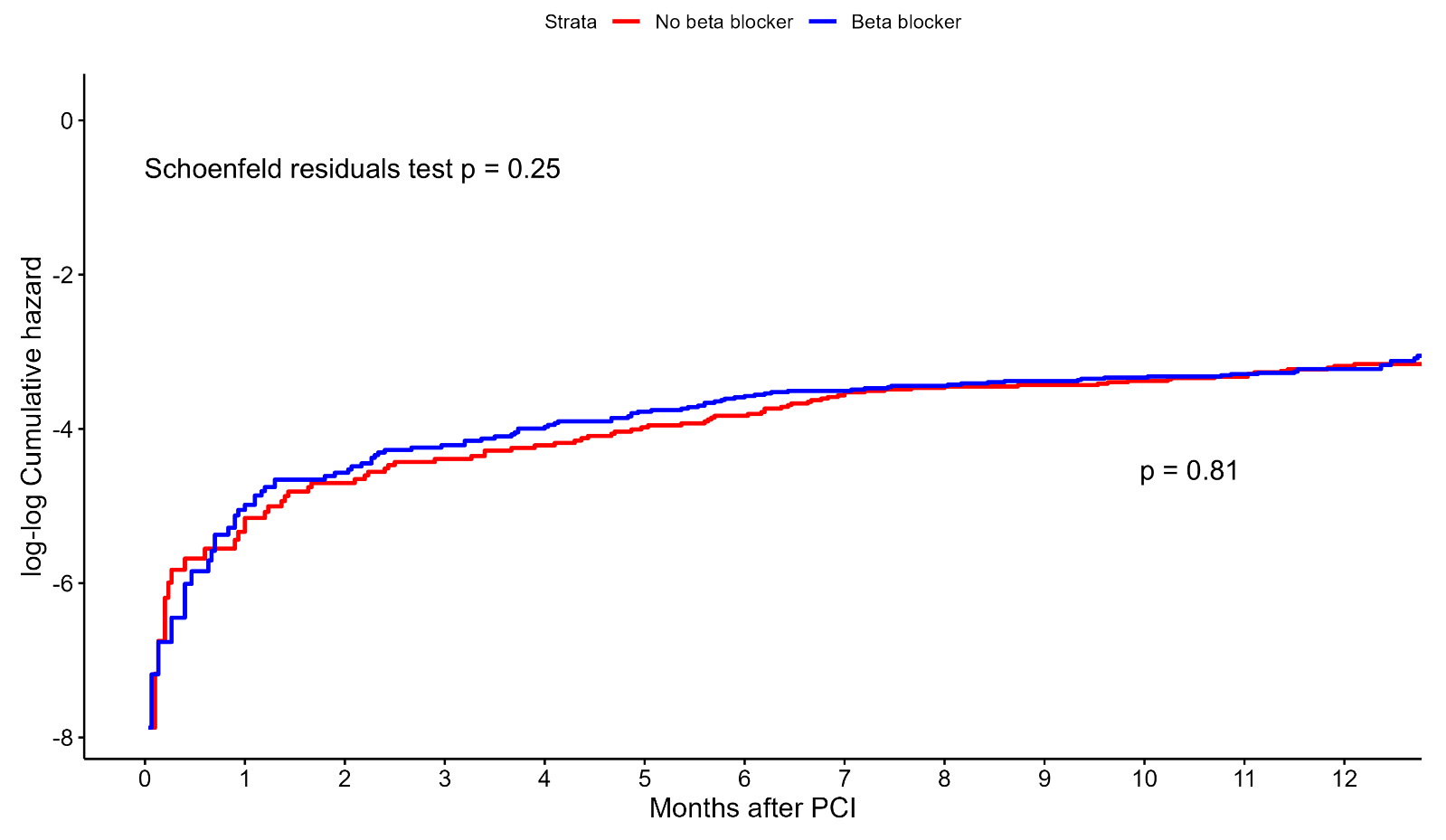
**

POCE, patient-oriented cardiac event

**Supplementary Table 1. Clinical outcomes at 1-year follow-up according to the use of beta blocker in the total population.**

|  | **Carvedilol** | **Bisoprolol** | **Nebivolol** | **No BBs** | **Carvedilol vs. Bisoprolol (Ref.)** | **P-value** | **Carvedilol vs. Nebivolol (Ref.)** | **P-value** | **Nebivolol (Ref.) vs. Bisoprolol** | **P-value** |
| --- | --- | --- | --- | --- | --- | --- | --- | --- | --- | --- |
|  | (N = 4,273) | (N = 2,800) | (N = 2,321) | (N = 2,633) | HR  (95% CI) |  | HR  (95% CI) |  | HR  (95% CI) |  |
| POCE, n (%) | 112 (2.6) | 108 (3.9) | 72 (3.1) | 89 (3.4) | 0.77  (0.59-1.01) | 0.055 | 0.58  (0.32-1.07) | 0.080 | 1.34  (0.85-2.10) | 0.211 |
| All-cause mortality, n (%) | 54 (1.3) | 46 (1.6) | 42 (1.8) | 43 (1.6) | 0.88  (0.59-1.31) | 0.528 | 0.96  (0.45-2.05) | 0.917 | 1.31  (0.68-2.54) | 0.422 |
| Any MI, n (%) | 46 (1.1) | 32 (1.1) | 27 (1.2) | 28 (1.1) | 1.07  (0.68-1.68) | 0.763 | 0.56  (0.19-1.63) | 0.283 | 0.67  (0.27-1.71) | 0.405 |
| Any revascularization, n (%) | 69 (1.6) | 63 (2.2) | 44 (1.9) | 50 (1.9) | 0.82  (0.58-1.15) | 0.249 | 0.36  (0.15-0.83) | 0.017 | 1.04  (0.559-1.82) | 0.900 |
| Cardiac death, n (%) | 25 (0.6) | 24 (0.9) | 22 (0.9) | 23 (0.9) | 0.79  (0.45-1.39) | 0.417 | 1.35  (0.45-4.03) | 0.587 | 1.95  (0.73-5.17) | 0.182 |
| Non-cardiac death, n (%) | 29 (0.7) | 22 (0.8) | 20 (0.9) | 20 (0.8) | 0.98  (0.56-1.70) | 0.931 | 0.69  (0.23-2.06) | 0.506 | 0.89  (0.35-2.25) | 0.806 |
| Admission for HF, n (%) | 41 (1.0) | 33 (1.2) | 33 (1.4) | 42 (1.6) | 0.94  (0.59-1.48) | 0.781 | 1.18  (0.53-2.62) | 0.693 | 0.93  (0.42-2.03) | 0.848 |

PS, propensity score; BB, beta blocker; HR, hazard ratio; CI, confidence interval; POCE, patient-oriented cardiac event; MI, myocardial infarction; HF, heart failure
